# Bilateral bifoveate retina in a human resembling lizard foveal architecture

**DOI:** 10.1080/13816810.2026.2679547

**Published:** 2026-05-28

**Authors:** Anil Kumar, Ashley M. Rasys, Brian P. Brooks, Mervyn G. Thomas

**Affiliations:** a Department of Ophthalmology, University Hospitals Derby and Burton NHS Foundation Trust, Derby, UK; b Ophthalmic Genetics and Visual Function Branch, National Eye Institute, National Institutes of Health, Bethesda, Maryland, USA; c Department of Ophthalmology, University Hospitals of Leicester NHS Trust, Leicester, UK; d Ulverscroft Eye Unit, School of Psychology and Vision Sciences, University of Leicester, Leicester, UK

**Keywords:** Fovea, foveal hypoplasia, bifovea, lizard, anolis

## Abstract

We describe a 45-year-old man with bilateral bifoveate retinas, an exceptionally rare human phenotype. Spectral-domain OCT demonstrated two distinct foveal pits per eye: a deep central fovea and a shallower temporal fovea with grade 1 foveal hypoplasia features. OCT-angiography revealed paired foveal avascular zones bilaterally. Unlike previously reported cases, which were predominantly unilateral and nonfunctional, both temporal foveae in this case show well-developed architecture closely resembling the bifoveate visual system of Anolis lizards. We propose this represents a rare partial failure of the spatial constraints that normally confine CYP26A1-mediated retinoic acid signaling to a single foveal locus in primates.

A 45-year-old man was referred for evaluation of an accessory parafoveal depression adjacent to the fovea. Visual acuity was 6/6 bilaterally with normal anterior segments. Fundoscopy revealed subtle temporal foveal depressions adjacent to the central foveae. Spectral-domain OCT demonstrated two distinct foveal pits in each eye: a deep central fovea and a shallower temporal fovea (Figure 1). The temporal fovea exhibited grade 1 foveal hypoplasia features, including continuation of inner retinal layers at the foveola, outer nuclear layer widening and outer segment lengthening (1, 2). OCT-angiography showed paired foveal avascular zones, with the temporal FAZ smaller than the central FAZ, reflecting incomplete vascular remodeling.

Bifoveate retinas in humans is exceptionally rare. To date, only three prior cases have been reported, termed “accessory fovea,” or “foveal pseudoduplication.” (3-5). These cases were predominantly unilateral, although one bilateral case has been described; all showed incomplete inner retinal displacement at the accessory site and were considered nonfunctional with respect to fixation or high-acuity visual processing. Our case represents an example of bilateral bifoveation with well-developed fovea architecture, resembling the bifoveate visual system of birds and reptiles, particularly *Anolis* lizards, which possess both deep central foveae and shallow temporal foveae with retained inner retinal layers (6-8).

These grade 1 hypoplasia characteristics (shallow pit, inner retinal layer continuation, outer segment lengthening, smaller FAZ) (1), mirror lizard temporal foveae, where retention of all retinal layers serves distinct binocular vision functions (6). This suggests a secondary zone of partial foveal specification undergoing incomplete developmental progression.

Recent genome-wide association study by Hunt et al. have identified retinoic acid metabolism, particularly involving the retinoic acid-degrading enzyme *CYP26A1*, along with cell-fate regulators, pigmentation genes, and extracellular matrix components (including collagens), as key determinants of human foveal pit formation (9). CYP26A1-mediated RA suppression is essential not only for patterning the human macula, but for specifying high-acuity retinal regions across vertebrate species more broadly and in retinal organoids (10-12). Complementary single-cell transcriptomic studies of the developing human retina demonstrate the importance of retinoic acid-depleted microenvironments in specifying cone-rich, high-acuity foveal regions (13). While the precise contribution of these pathways to the development of the lizard bifovea remains under active investigation, their conservation across vertebrates implies that the molecular program enabling bifoveal specialization is not absent in primates, it is suppressed. The paired foveal structures in our case suggest a rare partial failure of the spatial constraints that normally confine RA-mediated foveal specification to a single retinal locus.

The differential diagnosis includes perifoveolar thinning described in Alport syndrome, where focal temporal macular depressions have been reported (14). However, the bilateral symmetry, paired FAZs, preserved outer retinal specialization, and absence of systemic features support a diagnosis of true bifoveate morphology rather than degenerative thinning. We use the term “bifoveate retina”; as a morphological descriptor rather than a defined Mendelian entity; the phenotype is not currently cataloged in OMIM. The accessory temporal fovea exhibits features consistent with the foveal hypoplasia spectrum, which encompasses multiple genetically distinct OMIM-registered disorders.

This case expands the known spectrum of human foveal development and invites the tantalizing interpretation that the molecular programs encoding bifoveal architecture are not evolutionarily lost in primates, but latent, capable of rare reactivation, producing a human phenocopy of the bifoveate visual systems of birds and reptiles.

## Figures and Tables

**Figure 1.**
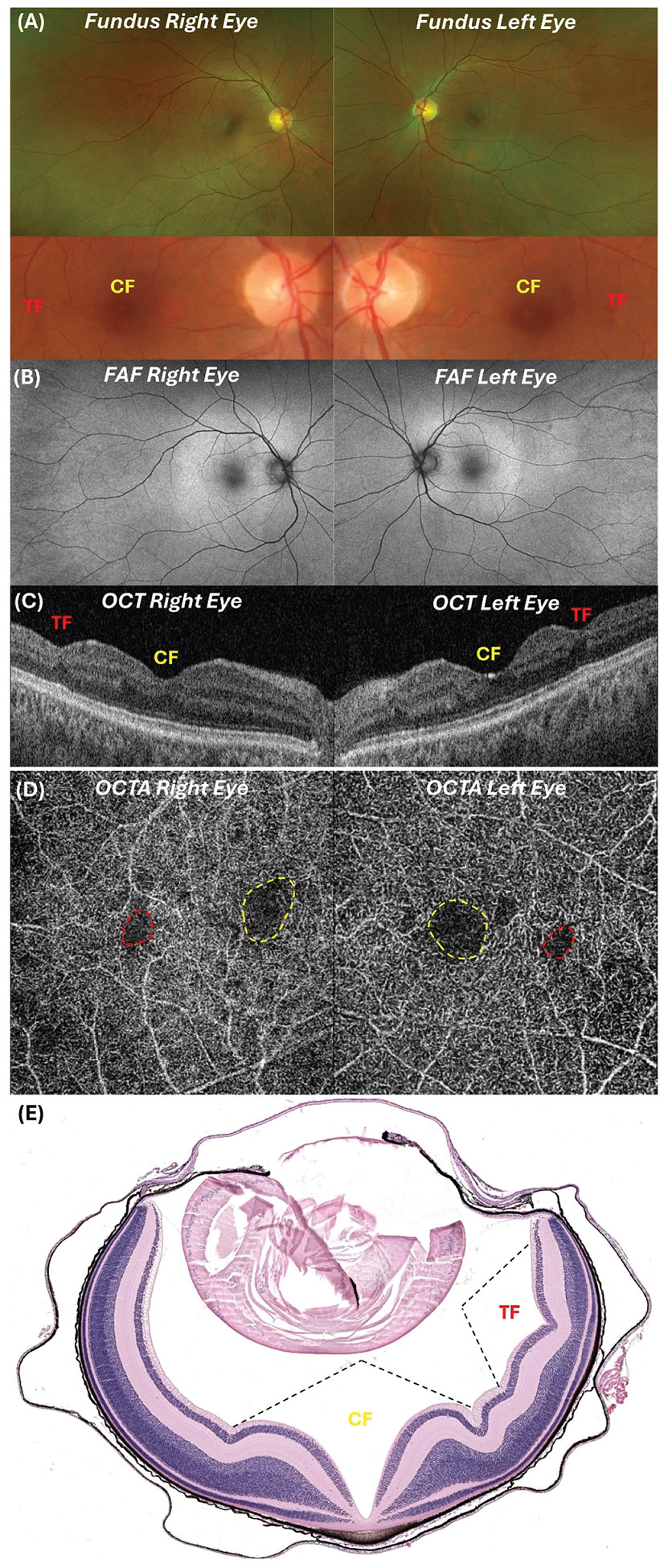
Bilateral bifoveate morphology with paired foveal avascular zones. (A) Fundus photos of right and left eyes, showing location of central (CF) and temporal fovea (TF) on magnified images. (B) Fundus autofluorescence images for both eyes. (C) Spectral-domain OCT of the right and left eyes demonstrating two distinct foveal pits per eye: a deep central fovea (CF, yellow) and a shallower temporal fovea (TF, red). The temporal foveae show persistence of inner retinal layers and reduced pit depth compared with the central foveae. (D) OCT angiography reveals paired foveal avascular zones in each eye, with the temporal FAZ smaller than the central FAZ. (E) Histological section of a bifoveate Anolis retina illustrating a deep central fovea (CF) and a shallower temporal fovea (TF) with retained inner retinal layers, demonstrating close architectural similarity to the human phenotype observed.
